# Fast-Acting Subperception Spinal Cord Stimulation for Persistent Spinal Pain Syndromes: Real-World Outcomes for up to 3 Years in an “As-Treated” Cohort from a Global Observational Study

**DOI:** 10.3390/jcm15197640

**Published:** 2026-10-02

**Authors:** Clark Metzger, Blake Hammond, Richard Ferro, Jose Paz-Solis, Georgios Kyriakopoulos, Philippe Rigoard, Ashish Gulve, Jose Emilio Llopis, Pablo Andrade, Sarah Love-Jones, Jan Willem Kallewaard, Jan Vesper, Stephen Pyles, Simon Bayerl

**Affiliations:** 1 Orthopedic Surgery Department, HCA Florida West Orthopedic Specialists, Pensacola, FL 32514, USA; 2 Interventional Pain Management Department, Multidisciplinary Pain Management Services, Okemos, MI 48864, USA; 3 Department of Neurosurgery, Hospital Universitario La Paz, 28046 Madrid, Spain; 4 Johanniter-Kliniken Hamm, 59063 Hamm, Germany; 5 Department of Neuro-Spine and Neuromodulation, Poitiers University Hospital, 86021 Poitiers, France; 6 Department of Anesthesiology and Pain Management, James Cook University Hospital, Middlesbrough TS4 3BW, UK; 7 Department of Anesthesiology and Pain Management, Hospital de la Ribera, 46600 Valencia, Spain; 8 Department of Stereotactic and Functional Neurosurgery, University Hospital Cologne, 50937 Cologne, Germany; 9 Department of Anesthesiology and Pain Management, Southmead Hospital, Bristol BS10 5NB, UK; 10 Department of Anesthesiology and Pain Management, Rijnstate Hospital, 6815 AD Arnhem, The Netherlands; 11 Department of Stereotactic and Functional Neurosurgery, Heinrich Heine University, 40225 Düsseldorf, Germany; 12 Interventional Pain Management Department, Florida Pain Clinic, Ocala, FL 34471, USA; 13 Department of Neurosurgery, Inter Neuro, 10629 Berlin, Germany

**Keywords:** fast-acting subperception therapy, pain relief, persistent spinal pain syndrome, spinal cord stimulation

## Abstract

**Background/Objectives:** Evidence on the effectiveness of spinal cord stimulation using fast-acting subperception therapy (FAST-SCS) in patients with persistent spinal pain syndrome (PSPS) remains limited. We evaluated clinical outcomes of FAST-SCS in patients with PSPS types 1 (i.e., non-surgical back pain) and 2 (i.e., failed back surgery syndrome). **Methods:** Our global, multicenter, retrospective, observational chart review included all consecutive adults with chronic low back and/or leg pain due to PSPS who received SCS with a multimodal device and reported using FAST-SCS at their latest follow-up. Available data from the patients’ medical files, documented by site personnel per their standard of care, were collected and analyzed. Pain intensity was assessed using the numerical rating scale (NRS); percent pain relief (PPR) was recorded where available. Main outcomes included changes in overall pain (NRS) compared to baseline, proportion of patients with ≥50% reduction in overall pain (NRS), proportion of patients with minimal pain (NRS ≤ 2), and high responder rates (PPR ≥ 80%). Data was analyzed for all PSPS patients at the last follow-up, with preliminary analysis of patients who completed 3-year assessments. Results in PSPS type 1 and 2 patients at the last follow-up are also presented. **Results:** In total, 424 patients (mean age 61.6 ± 13.5 years; 57% female; 61% PSPS type 2) were included. Mean baseline NRS was 8.0 ± 1.4. At the last follow-up (mean 1.6 ± 1.5 years post-implantation), NRS decreased by 5.3 points to 2.7 ± 2.3 (*p* < 0.0001), 77% of patients achieved ≥50% reduction in pain, 56% reported minimal pain, and 62% were high responders. Similar improvements were observed in the preliminary analysis of 104 PSPS patients who completed the 3-year follow-up, with ≥5-point reduction in the NRS, 77% responder rate, 60% patients with minimal pain, and 70% high responders. The analysis of PSPS type 1 and 2 populations, adjusted for baseline NRS and follow-up durations, showed comparable outcomes. **Conclusions:** In this global, observational, “as-treated” cohort, favorable real-world outcomes were observed in PSPS patients using FAST-SCS, supporting its use as an effective treatment option in chronic spinal pain management.

## 1. Introduction

Neuropathic pain represents a common and frequently disabling chronic pain state resulting from injury or dysfunction of the somatosensory nervous system [1,2,3]. Conventional pharmacological approaches often provide limited and incomplete analgesia, underscoring the need for complementary treatment options [4,5,6,7,8,9].

Spinal cord stimulation (SCS) has been used in clinical practice to manage chronic neuropathic pain since 1967. Low-frequency (<100 Hz), paresthesia-based stimulation (where patients feel a tingling sensation) activates dorsal column fibers that synapse with inhibitory GABAergic interneurons in the dorsal horn to modulate nociceptive transmission pathways, resulting in a clinically meaningful reduction in pain and functional improvements in appropriately selected patients [10,11,12,13].

For the first 30 years, SCS primarily managed patients suffering from chronic neuropathic pain after spinal surgery—previously defined as failed back surgery syndrome (FBSS) [14,15,16,17,18,19], now referred to as persistent spinal pain syndrome type 2 (PSPS type 2) [20]. For this indication, pain is typically lumbosacral with radicular and/or axial distribution, and patients mostly present with low back and/or leg pain. As a direct application of the gate control theory of pain, SCS treatment primarily targeted the radicular leg pain component. In fact, the first randomized controlled study to compare paresthesia-based SCS to conventional medical management in PSPS type 2 patients excluded those with predominant back pain and defined the primary endpoint as the radicular leg pain responder rate [21,22].

Over the years, greater understanding of both neural targets and neuromodulation mechanisms of action has led to the development of novel modalities using sub-threshold stimulation [23,24,25,26,27,28]. Initially designed with high-frequency (1000–10,000 Hz) continuous and/or burst patterns, these subperception paradigms offered opportunities to improve clinical outcomes [23,24,27] and/or sustain benefits over the long term [27,29,30,31,32]. For some SCS devices, access to different programmable stimulation options allows patients to decide for themselves which modality works best for them. Interestingly, the patient’s decision to use a particular waveform may reflect criteria other than just the pain reduction, including perception of comfort and ease of device management (e.g., charging burden) [33,34]. Thus, patients may prefer to use a modality that achieves clinically meaningful pain relief and offers the desired perception while being easy to use on a daily basis.

More recently, fast-acting subperception therapy (FAST-SCS) was developed. Like paresthesia-based SCS, FAST-SCS operates at a low frequency (90 Hz) but uses biphasic pulses with active recharge and precise neural dosing programming at sub-threshold stimulation levels (~30% perception threshold) to activate the surround inhibition mechanism and selectively engage the inhibitory circuit operation, thus further reducing transmission of pain to the brain [35,36,37,38]. While targeting the same neural elements as paresthesia-based SCS (mediated via myelinated Aβ fibers of the dorsal column), FAST-SCS preferentially targets the inhibitory surround fibers while at the same time limiting activation of excitatory center fibers.

Besides offering new programming options, robust clinical evidence for subperception modalities has demonstrated its effectiveness in reducing the axial low back pain component in PSPS type 2 patients [23,39,40]. In addition, multiple randomized clinical trials over the last 5 years have confirmed that when compared to conventional medical management, SCS can significantly improve chronic predominant low back pain with or without leg pain in PSPS patients without prior spine surgery (i.e., patients with PSPS type 1) [20,41,42,43]. Consequently, and in the context of recent mechanistic-based low back pain phenotyping efforts (nociceptive, neuropathic, or nociplastic), clinical applications of SCS for chronic low back and/or leg pain may now consider both types of appropriately selected PSPS patients, with or without prior spine surgery [44,45,46] when conservative treatments have failed and no surgical option is available.

Since its first use in clinical practice in 2021, multiple publications have assessed FAST-SCS clinical outcomes using various study designs [43,47,48,49,50,51] and confirmed the effectiveness of FAST-SCS. However, current evidence has only partially answered questions related to its use in long-term implanted patients, as well as outcomes in specific low back and/or leg pain patient populations such as PSPS types 1 and 2, which today constitute the majority of SCS indications worldwide [8,52,53]. Additional evidence is needed to further assess and better characterize real-world clinical outcomes of FAST-SCS in specific chronic pain indications.

The goal of our chart review was to determine clinical outcomes in a large consecutive cohort of PSPS patients with chronic low back and/or leg pain who reported using FAST-SCS as per standard of care practices across multiple centers in Europe and the US. We present here the outcomes observed in an “as-treated”, preferential usage cohort, including preliminary results for up to 3 years of follow-up and comparative response in PSPS types 1 and 2 patients.

## 2. Materials and Methods

### 2.1. Study Design

This global observational study, sponsored by Boston Scientific, was designed to characterize real-world clinical outcomes with the use of multimodal SCS systems for the treatment of chronic pain. The study is a retrospective, multicenter, de-identified patient data review and includes consecutively enrolled subjects whose data can be analyzed via multiple independent cohorts to evaluate clinical outcomes in different subgroups (ClinicalTrials.gov identifier NCT01550575). Multimodal systems offer possibilities to personalize SCS programming to each individual patient according to their needs, chronic pain condition, and/or preferences; accordingly, paresthesia-based stimulation, subperception options and combination therapies are available. To better characterize the effects of FAST-SCS, our cohort analyzed the data from all patients with chronic low back and/or leg pain due to PSPS who reported using this modality at the time of their latest follow-up. Data was obtained from de-identified medical records documented across 13 centers throughout Europe and the US and analyzed. Each site obtained Ethics Committee approval for the study, which was conducted in accordance with Good Clinical Practice (ISO14155) guidelines and the Declaration of Helsinki [54]. All patients provided informed consent as per local regulatory requirements.

### 2.2. Study Participants

All patients eligible for SCS whose indications were compliant with the “directions for use” labeling of the device and local regulations were included. There were no exclusion criteria, as per the study protocol. Each center applied their standard practice to decide which modality/ies patients were exposed to during the SCS trial and whether to adjust the initial programming during routine follow-up visits after permanent implantation of the pulse generator. Using the global study database, our cohort consisted of chronic pain patients (aged ≥18 years at baseline) who had been implanted with a multimodal SCS system (Boston Scientific Spinal Cord Stimulation Systems, Valencia, CA, USA) for the treatment of PSPS type 1 or type 2 and who reported using the FAST-SCS modality as a preferred treatment at the time of their latest follow-up visit.

### 2.3. Outcome Measures

Prior to patient enrollment, clinical assessments and questionnaires had been administered and documented in patients’ medical files by the site personnel per standard practice, without involvement of the study sponsor. Participating sites used available and previously documented data from their patients’ medical files (including pre- and post-implantation visits) to complete the study electronic case report forms (CRFs). The global database was used by the study sponsor to extract the data related to the cohort of interest (i.e., PSPS patients, FAST usage at last follow-up) using the corresponding completed CRFs (medical history and programming forms).

Demographic information, pain location, and surgical history were recorded. The numerical rating scale (NRS, scored from 0 = no pain to 10 = worst pain) was used to assess pain intensity (mild pain, score ≤3; moderate pain, score 4–6; severe pain, score ≥7). The Percent of Pain Relief (PPR) questionnaire was used to evaluate how much pain relief patients reported (0–100%). Primary outcomes included the change in the overall NRS pain score compared to baseline NRS and the proportion of patients who reported ≥50% reduction in NRS (i.e., responder rate). In addition, the percentages of patients with “minimal pain” (overall NRS pain score ≤2) and high responders (PPR ≥ 80%) were calculated to reflect the proportion of patients with profound pain relief. When documented in patients’ medical files and reported in the study case report forms, functional disability assessments using the Oswestry Disability Index (ODI) and health-related quality of life (EuroQol 5-dimension 5-level visual analog scale, EQ5D-5L VAS) were also analyzed. Finally, patient-reported “preferred SCS modality” was documented. For this specific cohort, all patients who had indicated using FAST-SCS at their last follow-up visit were included in the analysis. The study protocol did not enable the use of chart review data to collect adverse events information.

### 2.4. Statistical Analysis

The study sponsor conducted the statistical analysis of the cohort and produced statistical reports describing changes in clinical endpoints compared to baseline. Because of the real-world and retrospective nature of the study, which included sites from multiple countries with different patient management and clinical assessment practices, the type and amount of datapoints could vary and fluctuate over time. As a minimal requirement, only cases with documented pain scores were considered for analysis. Data collected from the last available follow-up were analyzed for all patients. A logistic analysis was conducted to assess the association of various baseline characteristics with SCS response (i.e., ≥50% pain relief) at the last follow-up. Outcomes were analyzed for PSPS types 1 and 2 subgroups, and a Generalized Linear Model was used to compare pain scores at the last follow-up when adjusted to the duration of follow-up and to the baseline pain score. Finally, a longitudinal analysis was performed for all patients who had reached a 3-year follow-up. For these patients, all clinical assessments that had been documented within a predefined time window around 1, 2, and 3 years after device implantation were analyzed. The normality of the change in NRS score was confirmed by a Kolmogorov–Smirnov Test. Means and standard deviations were determined for demographic data and NRS scores, while the score distribution was calculated for NRS and PPR. To calculate whether the mean reduction in baseline pain was greater than 0, a paired *t*-test with a two-sided 0.05 significance level was used. Continuous variables are presented as mean ± standard deviation, while categorical variables are presented as frequency (percentage). All statistical analyses were performed using SAS System Version 9.3 software or above (SAS Institute Inc., Cary, NC, USA).

## 3. Results

### 3.1. Patient Population

In total, 424 consecutive patients with PSPS (mean age 61.6 ± 13.5 years, 57% females) enrolled from 13 different sites in Europe and the US were deemed eligible for this FAST-SCS cohort, and their documented data was included in our analysis. All patients had been implanted with either percutaneous (70%) or surgical (30%) SCS leads, and an SCS device capable of multiple stimulation waveforms (Boston Scientific Neuromodulation, Valencia, US). In all subjects, FAST-SCS was reported as their preferred therapy at the time of the most recent follow-up visit.

PSPS type 1 had been diagnosed in 39% of patients (*n* = 166) and PSPS type 2 in 61% (*n* = 258). For the latter group, the most frequent prior spine surgeries comprised vertebral fusion (61%, *n* = 157), lumbar decompression procedures (43%, *n* = 111), and discectomy (22%, *n* = 57). Prior to SCS therapy, patients had severe pain (average overall NRS pain score 8.0 ± 1.4; N = 424). Similar baseline overall pain scores were reported in PSPS type 1 (8.1 ± 1.3, *n* = 166) and type 2 patients (7.9 ± 1.5, *n* = 258) (Table 1).

### 3.2. Results in All PSPS Patients (N = 424)

**Figure 1:**
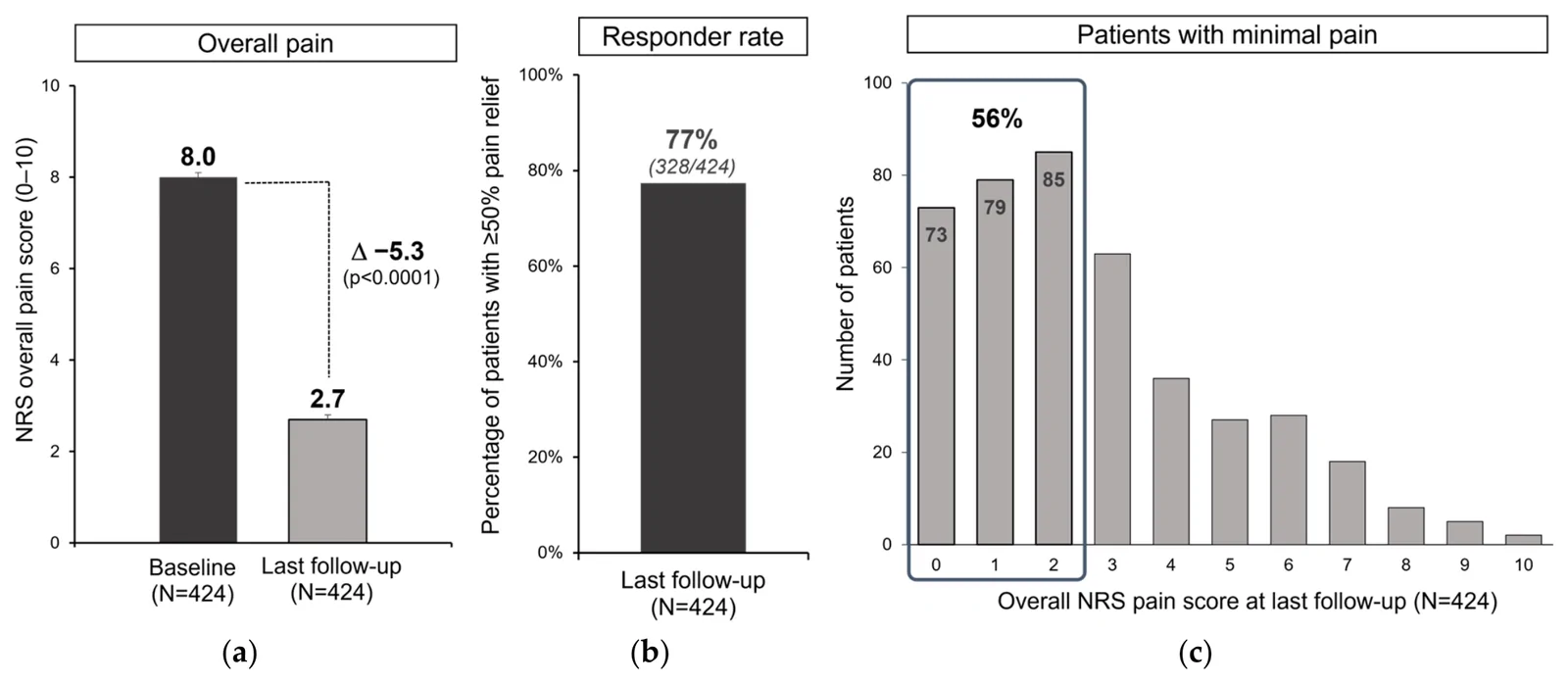
Results at last follow-up (mean 1.6 ± 1.5 years) in all persistent spinal pain syndrome patients (N = 424): (**a**) mean overall NRS pain score; (**b**) responder rate (proportion of patients with overall NRS pain score reduction ≥50%); (**c**) distribution of overall NRS pain scores and proportion of patients with minimal pain at last follow-up (NRS ≤ 2). Abbreviations: NRS, numerical rating scale.

The average follow-up duration was 1.6 ± 1.5 years after implantation of the SCS system (median follow-up duration 1.2 years). During this time, a significant 5.3-point decrease in the overall NRS pain score was observed from baseline (8.0 ± 1.4) to the last follow-up (2.7 ± 2.3; *p* < 0.0001) (Figure 1a). At the last follow-up, the proportion of patients who experienced ≥50% reduction in their overall NRS pain score was 77.4% (328/424) (Figure 1b). Minimal pain (overall NRS ≤ 2) was reported by 56% (237/424) of patients (Figure 1c). Finally, the PPR data was documented in 69% (292/424) of patients; of these, 62% (180/292) of patients were high responders (PPR ≥ 80%).

A logistic analysis was performed to examine the association of responder rate at last follow-up and independent variables such as age, sex, diagnosis (PSPS type 1 or 2) and baseline NRS. None of these evaluated covariates demonstrated a clinically meaningful predictive effect.

Functional disability (ODI score) and health-related quality of life (EQ5D-5L VAS score) were only reported in 20% (*n* = 83) and 23% (*n* = 98) of patients, respectively. ODI improved by −26.4 ± 22.9 points when compared to baseline (*p* < 0.0001, *n* = 64) and the mean ODI score at the last follow-up was 34.7 ± 20.2 (*n* = 77), corresponding to moderate disability. Overall health (EQ5D-5L VAS) also significantly improved from 28.5 ± 17.6 at baseline to 76.3 ± 21.5 at the last follow-up (+52.2 ± 29.8; *p* < 0.0001; *n* = 95).

**Figure 2:**
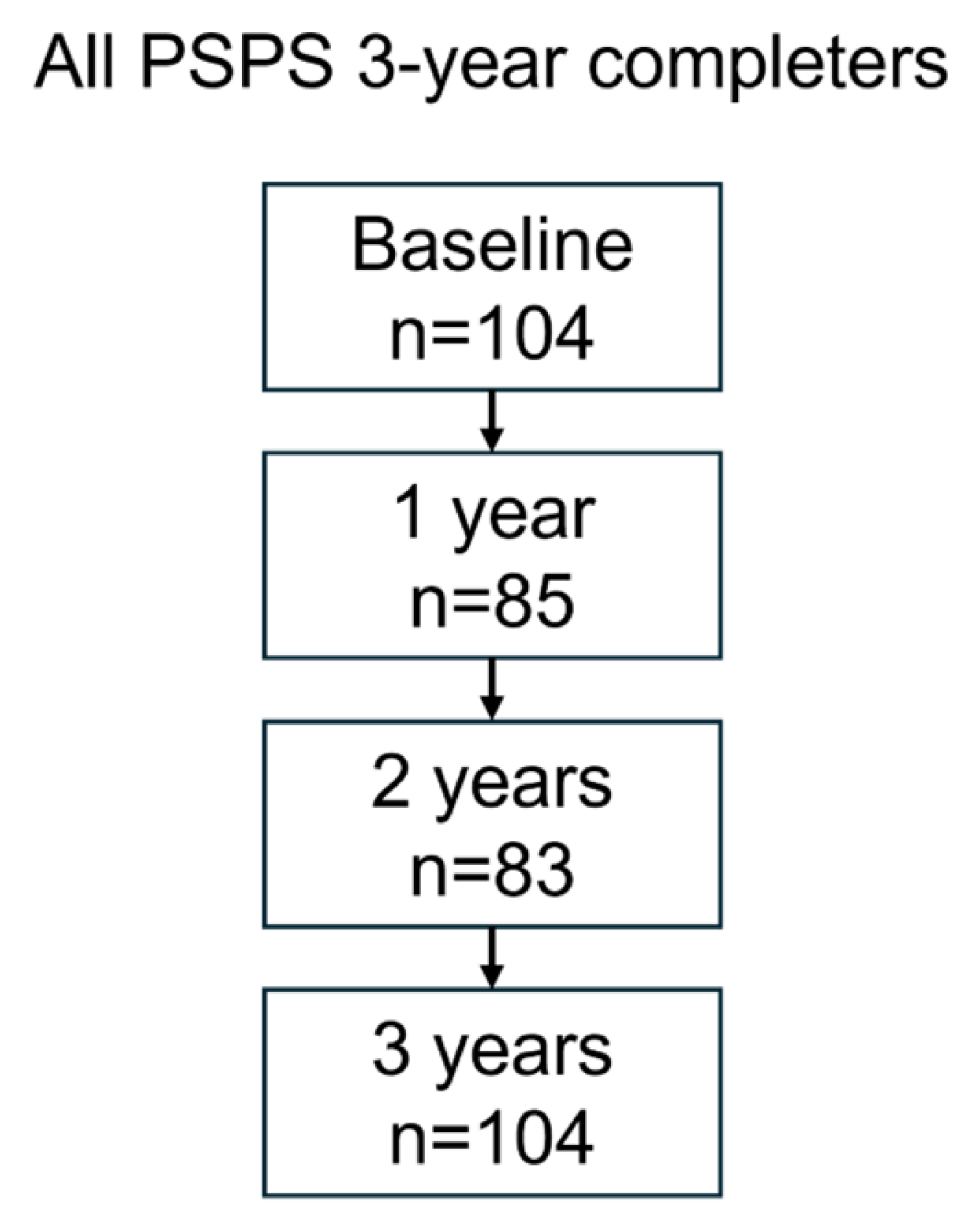
Flow diagram for 3-year follow-up completers in patients with persistent spinal pain syndrome (PSPS). All datasets analyzed at 1-, 2-, and 3-year follow-ups belong to the same population of 3-year follow-up completers (*n* = 104).

**Figure 3:**
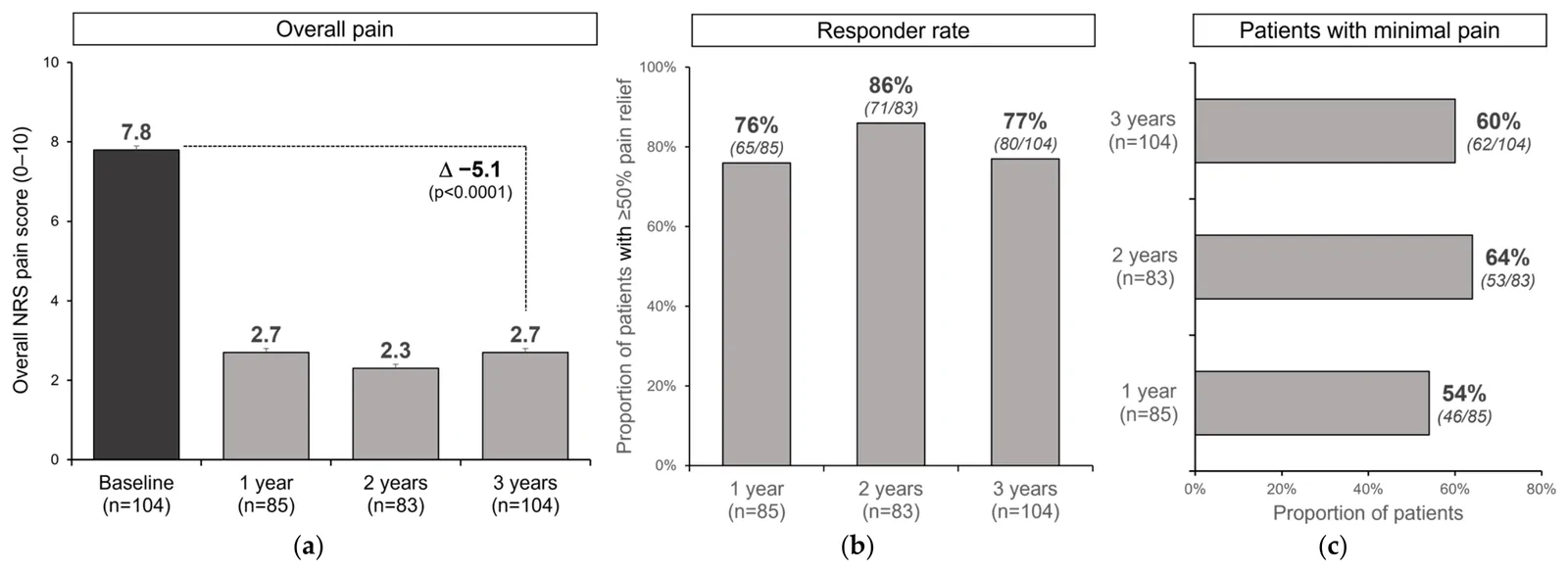
Pain relief at the 1-, 2-, and 3-year follow-ups for persistent spinal pain syndrome patients who completed the 3-year follow-up (*n* = 104): (**a**) overall NRS pain score; (**b**) responder rate (overall NRS reduction ≥ 50%); (**c**) proportion of patients with minimal pain (NRS ≤ 2). Abbreviations: NRS, numerical rating scale.

Finally, the preliminary analysis of outcomes at 3 years was possible in a subset of 104 PSPS patients who had been implanted for this length of time at their last follow-up. A longitudinal analysis was conducted with all documented assessments at the 1-, 2-, and 3-year follow-up visits for this 3-year completer population (Figure 2). Compared with baseline, the overall NRS pain had decreased significantly by 5.1, 5.4, and 5.1 points at the 1-, 2-, and 3-year follow-up visits, respectively (*p* < 0.0001) (Figure 3a). At these timepoints, a ≥50% reduction in the overall NRS pain score was reported by 76% (65/85), 86% (71/83), and 77% (80/104) of patients (Figure 3b). Minimal pain (overall NRS ≤ 2) was reported by 54% (46/85), 64% (53/83), and 60% (62/104) of patients (Figure 3c). Of the 84 patients who completed the PPR questionnaire at 3 years, 70% (59/84) were high responders (PPR ≥ 80%). Health-related quality of life assessment was available for 53 patients at the 3-year follow-up. The EQ5D-5L VAS score improved from 23.3 ± 12.1 at baseline (*n* = 46) to 81.8 ± 18.0 at 3 years (*n* = 53) (+61.2 ± 20.3, *p* < 0.0001).

### 3.3. Results in PSPS Type 1 and Type 2 Patients

**Figure 4:**
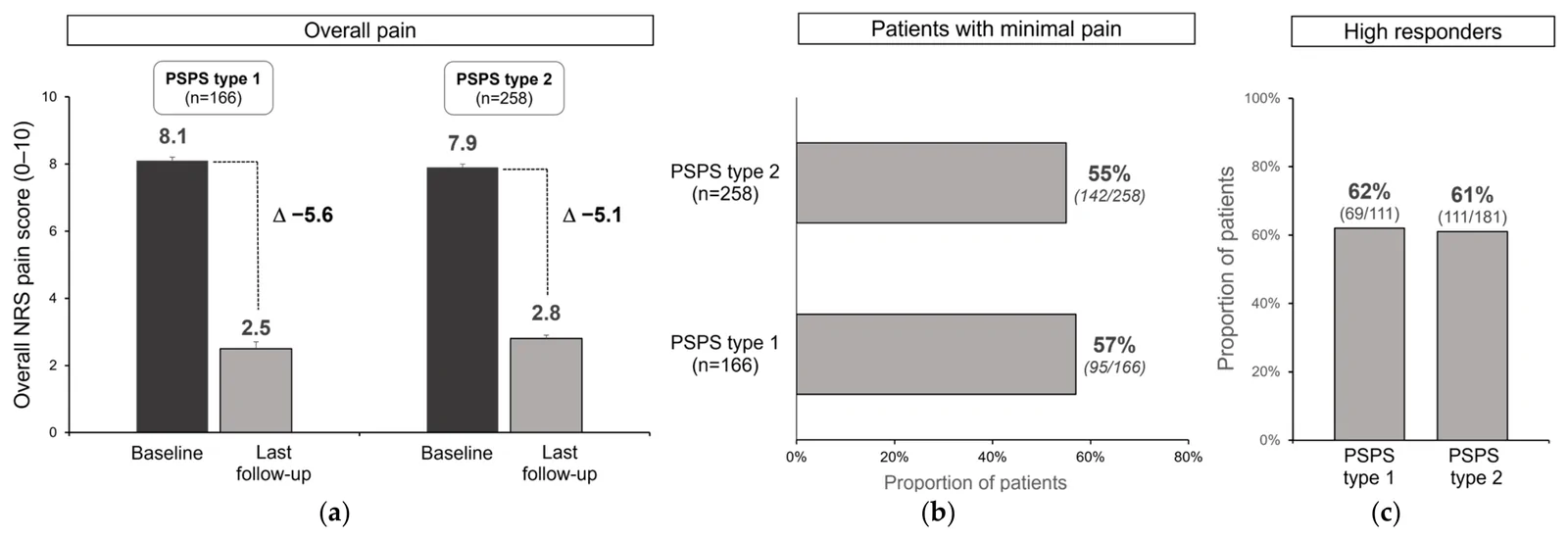
Results in PSPS type 1 (*n* = 166) and type 2 (*n* = 258) patients at the last follow-up (mean follow-up duration: 1.0 ± 1.1 year in PSPS type 1 patients; 2.0 ± 1.6 years in PSPS type 2 patients): (**a**) mean overall NRS pain score; (**b**) proportion of patients with minimal pain (NRS ≤ 2); (**c**) high responder (PPR ≥ 80%) rates. Abbreviations: NRS, numerical rating scale; PSPS, persistent spinal pain syndrome.

At the mean last follow-up visit after implant (1.0 ± 1.1 years in PSPS type 1; 2.0 ± 1.6 years in PSPS type 2), there was a significant reduction in the mean overall pain scores in PSPS type 1 (−5.6 ± 2.4 points; *p* < 0.0001) and type 2 patients (−5.1 ± 2.7 points; *p* < 0.0001) (Figure 4a). A ≥50% reduction in the overall pain score was observed at the last follow-up in 81% (135/166) of PSPS type 1 patients and 75% (193/258) of PSPS type 2 patients. Minimal overall pain (NRS ≤ 2) was reported by 57% (95/166) of PSPS type 1 and 55% (142/258) of PSPS type 2 patients at last follow-up (Figure 4b). At this timepoint, the PPR questionnaire had been collected in 111 of PSPS type 1 patients and 181 of PSPS type 2 patients. The PPR results show that the high responder rate (PPR ≥ 80%) was 62% (69/111) in PSPS type 1 and 61% (111/181) in PSPS type 2 patients (Figure 4c).

A Generalized Linear Model was used to compare the overall NRS pain score at last follow-up between PSPS type 1 and type 2 patients, adjusting for both baseline NRS values as well as follow-up durations. Results show that there was no difference in Least Square Mean between the PSPS type 1 and type 2 groups (*p* = 0.1263). Comparison of disability and health-related quality of life outcomes was not possible due to the lack of available data in PSPS type 1 subjects.

## 4. Discussion

Our global, real-world, multicenter review of FAST-SCS therapy in a large cohort of chronic pain patients with PSPS showed a significant and sustained >5-point reduction in overall pain scores. Of the 104 patients who had completed their 3-year visit, 60% had minimal pain (NRS ≤ 2) and 70% were high responders (≥80% pain relief). Our results are consistent with previous real-world findings with a 2-year follow-up [48,49,50], as well as a recently published prospective clinical study reporting 1-year outcomes [51]. Our data provides additional evidence into the long-term, real-world outcomes of FAST-SCS, using chart reviews from multiple sites within the US and Europe.

Our results also suggest that the FAST-SCS modality can provide significant and comparable pain relief in both PSPS types 1 and 2. The effectiveness of SCS, including paresthesia-based and subperception waveforms, has been investigated for PSPS type 2 patients using multiple study designs, including randomized controlled trials (RCTs) [21,22,23,39,40,55]. Systematic reviews, guidelines, and cost-effectiveness studies have confirmed the value of SCS for this challenging indication [8,18,56,57,58,59,60]. Besides PSPS type 2, several recent RCTs have specifically assessed the effectiveness of SCS in PSPS type 1 populations with predominant axial low back pain in comparison to conventional medical management. Results from these RCTs were consistent, with significant improvements observed in overall and low back pain [41,42,43]. Our observational data provide a real-world perspective of how FAST-SCS performs in these patients in standard clinical practice, which has not yet been assessed. While direct comparisons are not possible, our results suggest similar findings to those observed in the SOLIS US prospective multicenter RCT, which evaluated multimodal SCS for PSPS type 1 patients [43]. In this trial, the crossover outcomes at 1 year in over 100 PSPS type 1 patients showed that 86% were responders and 56% reported ≥80% pain relief, including patients using FAST-SCS therapy.

Our real-world pragmatic study has limitations. Our cohort of patients did not include all patients implanted with multimodal SCS devices and instead was limited to all consecutive patients who had reported preferential usage of FAST-SCS at their most recent follow-up visits. While this approach was chosen to characterize outcomes when using this modality in standard practice, the selection bias potentially represents the outcomes of FAST responders. Nevertheless, our data show that despite their preference for using FAST-SCS at their last follow-up, 23% of patients did not report ≥50% reduction in their overall pain score. This could be explained by the fact that some patients may not have been offered alternative options to choose from; hence, their “usage” of FAST-SCS may not necessarily be a real “preference”. Furthermore, even in patients who actually preferentially used FAST-SCS (i.e., they were able to choose from different options, as per the capabilities of their multimodal SCS device), evaluating the clinical response would still be valuable, as additional factors to pain reduction may have played a role in their choice. In 2016, Kriek et al. conducted a multicenter, double-blind, randomized, placebo-controlled crossover trial to compare the efficacy of multiple SCS waveform in complex regional pain syndrome patients [33]. Results showed similar pain relief across all “active” SCS modalities, yet a substantial proportion of patients (48%) preferred one specific waveform (preference for other programs ranged from 14% to 21%). This trend was explained by the fact that beyond pain relief outcomes alone, other patient preference criteria included perception comfort (paresthesia-based versus subperception), as well as device ease-of-use (i.e., charging burden). Our report focused on FAST-SCS usage, and additional analyses comparing this data to other cohorts using different modalities would be required to better understand how patients personalize their treatment when implanted with a multimodal device. Waveform usage may also evolve over time, and programming versatility may help adjust the SCS treatment to patients’ preferences as well as to the evolutive nature of their chronic pain conditions. The outcomes presented here reflect the effects of FAST-SCS at the time of their most recent follow-up (1.6 years post-implant on average), yet other waveforms may have been used since implantation; we did not analyze the evolution in SCS programming over time, which could also be an interesting topic to explore.

Another limitation in our study relates to attrition, as only 25% of the whole cohort (104/424) had a documented 3-year follow-up assessment, for various reasons: some patients had not yet reached their 3-year follow-up window (the median follow-up duration was 1.2 years for all 424 patients), some patients were lost to follow-up, while others had a 3-year visit but data was missing. However, our preliminary longitudinal analysis of all 104 patients with 3-year follow-up suggested that FAST-SCS could be a valuable contributor to the durability of SCS therapy, with a significant reduction in overall pain and sustained pain relief. Future research is expected to yield long-term follow-up data on a larger number of patients, as well as data on multidimensional outcomes, such as ODI and health-related quality of life.

Furthermore, the retrospective chart review design resulted in significant data gaps. Our study had no control group and no mandatory or systematically collected secondary outcomes (e.g., quality of life and functional disability). Data was gathered from pre-existing clinical evaluations that had been recorded in the patients’ medical files in each site, per their individual standard of care. Our analysis was therefore limited to those documented endpoints and the number of observations differed and fluctuated over time, resulting in data gaps and variations in follow-up timelines. For example, functional disability (ODI) and health-related quality of life data (EQ5D-5L VAS) assessments were reported in less than 25% of the total cohort at last follow-up, which limits the possibility of producing definitive conclusions about the multidimensional impact of FAST-SCS. This limitation can be mitigated with the recently published 1-year outcomes of FAST-SCS from a multicenter prospective study, which evaluated holistic clinical assessments [51]. Clinically significant improvements were reported across multiple dimensions, including physical functioning (ODI and objective mobility tests), quality of life, sleep quality, pain catastrophizing, and depression. The clinical relevance of future retrospective chart review cohort studies could be improved by limiting the database to patients whose medical files document multidimensional assessments.

Another limitation of our study is that per protocol, adverse events were not reported/entered into the database; thus, safety aspects could not be analyzed. However, previous prospective published reports related to similar multimodal devices and programming capabilities have presented the long-term safety profile of SCS in populations of chronic low back and/or leg pain patients [51,61]. In 2023, Rauck et al. described safety results for up to 3 years in a global prospective study of 1289 implanted patients [61]. Results showed a 3.5% annualized rate of device explants (all causes) and 1.1% due to inadequate pain relief.

Our results must be seen as a complementary and observational source of evidence, using real-world data from multiple standard clinical practices across different sites and geographies. Other studies have been conducted with more controlled study populations and/or designs [43,51]. Our real-world global analysis may contribute to a more comprehensive representation of the clinical evidence “spectrum” for FAST-SCS, which we believe is valuable for clinical practice applications. Future analyses are needed to confirm our findings and focus on developing evidence for longer-term follow-up and multidimensional clinical assessments.

## 5. Conclusions

Our global, multicenter, real-world observational study evaluated a cohort of more than 400 PSPS patients who preferentially used FAST-SCS at their last follow-up (mean 1.6 years). Favorable outcomes were observed, with ≥5-point reduction in their overall NRS pain score versus baseline, 77% responder rate, and 56% of patients with minimal pain (NRS ≤ 2). Similar results were observed in PSPS type 1 and type 2 patients. Preliminary long-term findings from 104 PSPS patients who had been assessed for up to 3 years after implantation showed that pain relief was maintained at the 1-, 2- and 3-year marks. FAST-SCS is one of several therapeutic options offered by multimodal devices, and our results suggest that it may contribute to the optimization and maintenance of SCS outcomes.

## Figures and Tables

**Table 1:** Baseline characteristics.

	All Patients	PSPS Type 1	PSPS Type 2
Number of subjects	424	166	258
Age—mean (SD) *n*	61.6 (13.5) 415	62.8 (13.9) 163	60.9 (13.2) 252
Sex (female)—% (*n*/N)	57 (241/424)	60 (100/166)	55 (141/258)
Prior spine surgeries (multiple procedures allowed)			
Vertebral fusion—% (*n*/N)	37 (157/424)	N/A	61 (157/258)
Lumbar decompression—% (*n*/N)	26 (111/424)	N/A	43 (111/258)
Discectomy—% (*n*/N)	13 (57/424)	N/A	22 (57/258)
Percutaneous leads—% (*n*/N)	70 (289/410)	71 (116/164)	70 (173/246)
Surgical leads—% (*n*/N)	30 (121/410)	29 (48/164)	30 (73/246)
Overall pain (NRS)—mean (SD) *n*	8.0 (1.4) 424	8.1 (1.3) 166	7.9 (1.5) 258
ODI—mean (SD) *n*	57.4 (16.2) 83	59.2 (14.1) 13 ^a^	57.1 (16.6) 70
EQ5D-5L VAS—mean (SD) *n*	28.5 (17.6) 98	52.8 (25.0) 4 ^a^	27.4 (16.7) 94
Follow-up duration (years)—mean (SD) *n*	1.6 (1.5) 424	1.0 (1.1) 166	2.0 (1.6) 258

^a^ Not analyzed due to limited number of documented assessments. Abbreviations: EQ5D-5L VAS, EuroQol 5-dimension 5-level visual analog scale; ODI, Oswestry Disability Index; N/A, not applicable; NRS, numerical rating scale; PSPS, persistent spinal pain syndrome; SD, standard deviation.

## Data Availability

The data, analytic methods, and study materials for this clinical study will be made available to other researchers in accordance with the Boston Scientific Data Sharing Policy: https://www.bostonscientific.com (accessed on 19 June 2026).

## Abbreviations

The following abbreviations are used in this manuscript:

CRF	Case report forms
EQ5D-5L	EuroQol 5-dimension 5-level visual analog scale
FAST	Fast-acting subperception therapy
FBSS	Failed back surgery syndrome
NRS	Numerical rating scale
ODI	Oswestry Disability Index
PPR	Percent of pain relief
PSPS	Persistent spinal pain syndrome
RCT	Randomized controlled trial
SD	Standard deviation
SCS	Spinal cord stimulation
VAS	Visual analog scale
